# Assessing 3-D Spatial Extent of Near-Road Air Pollution around a Signalized Intersection Using Drone Monitoring and WRF-CFD Modeling

**DOI:** 10.3390/ijerph17186915

**Published:** 2020-09-22

**Authors:** Seung-Hyeop Lee, Kyung-Hwan Kwak

**Affiliations:** 1 Department of Environmental Science, Kangwon National University, Chuncheon 24341, Korea; SeungHyeop@kangwon.ac.kr; 2 School of Natural Resources and Environmental Science, Kangwon National University, Chuncheon 24341, Korea

**Keywords:** near-road air pollution, 3-D spatial extent, signalized intersection, morning rush hour, drone monitoring, WRF-CFD modeling

## Abstract

In this study, we have assessed the three-dimensional (3-D) spatial extent of near-road air pollution around a signalized intersection in a densely populated area using collaborating methodologies of stationary measurements, drone monitoring, and atmospheric dispersion modeling. Stationary measurement data collected in the roadside apartment building showed a substantial effect of emitted pollutants, such as nitrogen oxides (NO_x_), black carbon (BC), and ultrafine particles (UFPs), especially during the morning rush hours. Vertical drone monitoring near the road intersection exhibited a steeper decreasing trend with increasing altitude for BC concentration rather than for fine particulate matter (PM_2.5_) concentration below the apartment building height. Atmospheric NO_x_ dispersion was simulated using the weather research and forecasting (WRF) and computational fluid dynamics (CFD) models for the drone measurement periods. Based on the agreement between the measured BC and simulated NO_x_ concentrations, we concluded that the air pollution around the road intersection has adverse effects on the health of residents living within the 3-D spatial extent within at least 120 m horizontally and a half of building height vertically during the morning rush hours. The comparability between drone monitoring and WRF-CFD modeling can further guarantee the identification of air pollution hotspots using the methods.

## 1. Introduction

The urban proportion of the global population is 55% and is expected to increase to 68% by 2050 [1]. Owing to the high population densities in urban areas, public use facilities, such as schools and parks in residential areas, have become closer to roads with increasing traffic. As a result, residents’ exposure to harmful air pollutants emitted by on-road mobile sources has become a serious health and environmental problem [2,3]. Air pollutants emitted from vehicles, such as nitrogen oxides (NO_x_), carbon monoxide (CO), black carbon (BC), and ultrafine particles (UFPs) with a diameter less than 100 nm, have caused various environmental diseases such as respiratory diseases, stunted growth in children, and cardiovascular diseases [4,5].

Environmental diseases caused by air pollutants are more likely to occur in air pollution hotspots where there are many pedestrians and mobile sources intensively [6,7]. Signalized road intersections are representative hotspots of air pollution in urban areas. Because frequent stops, accelerations, and decelerations of vehicles at traffic signals occur around busy intersections, signalized road intersections have a high risk of exposure to air pollutants emitted by vehicles, with more than 20% exceeding the average CO and NO_x_ emissions compared to roundabouts [8,9,10]. In addition, high-rise buildings near signalized intersections can weaken air flow and then worsen air quality [11,12].

Traffic-related air pollutants such as NO_x_ and UFPs can be reduced as the distance from the intersection increases by up to a few hundred meters in an open space [13], complex urban areas [14,15], and urban parks [16]. The horizontal gradient of pollutant concentration with increasing distance from the road is determined by road geometries and atmospheric environment such as wind speed and direction with respect to the road axis as well as on-road pollutant emissions [17]. A few previous studies conducted measurements on roadside high-rise buildings to investigate the vertical concentration profiles of traffic-related air pollutants. Major pollutants such as PM_10_, PM_2.5_(particulate matter with a diameter less than 10 μm and 2.5 μm, respectively) NO_2_, CO_2_, and CO emitted from the adjacent roads were gradually reduced as the measurement height increased because of vertical mixing [18,19]. To overcome the spatial limitations of vertical distribution measurements based on fixed structures such as towers and buildings, mobile measurements using a drone have recently been attempted by many researchers. A previous study [20] measured the vertical distribution of a particle number concentration using a drone near a highway in Australia and observed near-road air pollution up to at an approximately 40 m altitude. In addition, another previous study [21] measured the vertical PM_2.5_ distributions in the middle of roads using a drone, and found that these concentrations remained constant within a 35 m altitude and began to decrease above the altitude because of the effect of roadside trees.

Although air pollutant concentrations near hotspots such as road intersections show high spatiotemporal variabilities, simultaneous measurement of these concentrations at fixed locations to demonstrate the three-dimensional (3-D) spatial distribution is extremely challenging, especially in urban areas. To solve this problem, a computational fluid dynamics (CFD) model that simulates the airflow and pollutant distribution at road and building scales has been utilized [22,23,24]. The 3-D spatial distribution of near-road air pollution around hotspots is likely to be derived by the airflow around buildings, such as recirculating flows between roadside buildings and channeling flows inside building arrangements [25,26,27]. The peak concentration locations in a street canyon between buildings were substantially different within a 30 m altitude from the ground depending on the building geometries (i.e., height and symmetry) on both sides [28].

To provide realistic meteorological inputs for the inflow boundary conditions of the CFD model, the weather research and forecasting (WRF) model coupled to the CFD model is one of the several reliable methodologies [29]. A WRF-CFD modeling study [30] confirmed that the simulation accuracy of simulation results using the coupled WRF-CFD model improved compared to that of simulation results using the CFD model only. The high spatiotemporal resolution of meteorological input data, such as 3-D air temperature, wind components, and turbulent kinetic energy fields, with an approximately 1 km resolution is the essential merit of the coupled WRF-CFD model [31]. The coupled WRF-CFD modeling approach provides accurate meteorological inputs from a mesoscale model in simulating realistic urban air quality at a high resolution [32].

On-site measurement and high-resolution numerical simulations are complementary methods used to identify the spatiotemporal variation near air pollution hotspots in urban areas. Although a high-resolution numerical simulation is beneficial for providing detailed identification of spatiotemporal variability, an on-site measurement is essential for verifying and improving the reliability of numerical simulation results. However, it is still challenging to find the comparability between the smoothed concentration fields simulated using a numerical model and the instantaneous concentration fields measured by a mobile monitoring instrument. In this study, we use collaborating methodologies of stationary and mobile monitoring together with the coupled WRF-CFD modeling to quantitatively assess the 3-D spatial extent of near-road air pollution near an intersection in an urban residential area. Section 2 provides the methodology, Section 3 presents and discusses the results, and Section 4 concludes this paper.

## 2. Methods

### 2.1. Study Site and Period

**Figure 1:**
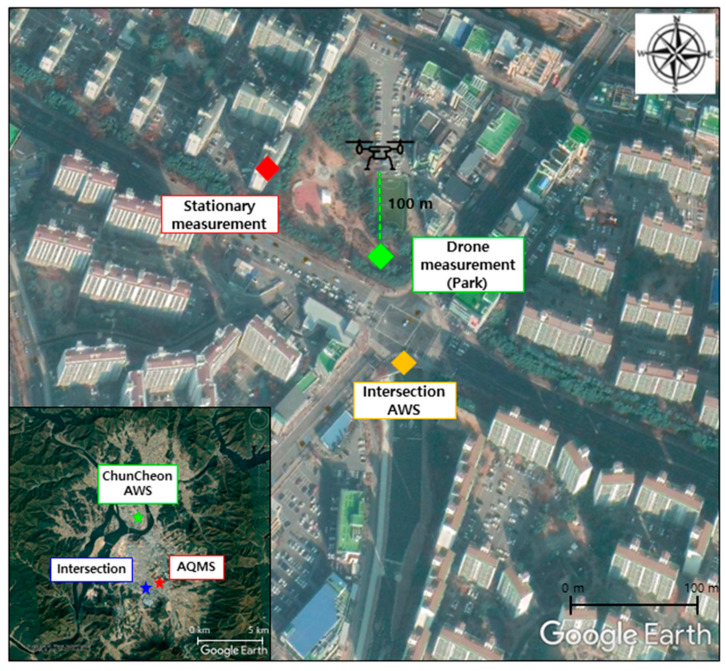
Measurement locations near the Toegye Intersection in Chuncheon, Gangwon Province, Republic of Korea. The map at the left bottom corner shows the entire Chuncheon basin. The Chuncheon AWS (automatic weather station) and the AQMS (air quality monitoring station) are located at approximately 5 km north and 1 km northeast of the intersection, respectively.

The site of interest is a signalized intersection in a residential area in Chuncheon, Gangwon Province in the Republic of Korea (37.88° N, 127.73° E, 105 m a.s.l. (i.e., above sea level)). Chuncheon is a medium-sized city located in an inland basin near a large river, with a population of approximately 280,000. Toegye Intersection surrounded by several apartment complex of 18 floors or less is the signalized intersection where six-lane roads in three directions intersect with a four-lane road in one direction. Because it is located on the road entering from the city center to the highway, the intersection is busy, containing significant traffic that consists of express buses and heavy-duty vehicles as well as passenger cars. An urban park is located at the northern side of the intersection, and a small stream running from south to north crosses the urban park (Figure 1).

The two periods of interest were on four consecutive weekdays from 12 to 15 March 2019 (P1) and on five consecutive weekdays from 1 to 5 April 2019 (P2). The drone measurements were conducted in the urban park at approximately 50 m north of the center of the intersection. The stationary measurements were conducted on the 11th floor (i.e., 30 m altitude above the ground) of an apartment building at approximately 150 m northwest of the center of the intersection. Two measurement locations were placed at the downwind side of the intersection when a southerly wind prevailed. From 10 min before every drone measurement, we recorded traffic videos of the intersection for two cycles of traffic signals (i.e., 320 s). Then, the traffic volumes were manually counted from the recorded video. Chuncheon automatic weather station (AWS), approximately 5 km North of the Toegye Intersection, is chosen for background meteorological observation data. In addition, AWS instruments were additionally installed on the sidewalk to the south of the Toegye Intersection for roadside meteorological observation data, such as temperature, relative humidity, wind direction, and wind speed. The air quality monitoring station (AQMS) data were obtained for urban background concentrations of air pollutants (i.e., PM_2.5_, NO_2_, O_3_).

### 2.2. Drone and Stationary Measurements

The drone measurements were performed during the morning rush hours (8–9 a.m.) on selected three days of 12, 14, and 15 March 2019 (P1D), and on another selected three days of 1, 2, and 3 April 2019 (P2D). The drone used in this study was an octocopter drone (DJI A2) with rotary wings that can hover at a certain altitude. The total width and height are 105 and 50 cm, respectively, and the flight time is approximately 10 min with a carrying capacity of 2 kg or more [33]. Meteorological (Imet-XQ_2_ for air temperature (Temp) and relative humidity (RH)), particulate matter (AM520 for PM_2.5_ and MA200 for BC), and gaseous (Aeroqual 500 for O_3_) sensors were used for drone measurements. These are small instruments that do not exceed 620 g per device (Table 1). The inlets connected to AM520 and MA200 to measure PM_2.5_ and BC were installed approximately 20 cm above the main body of drone to minimize the effect of turbulence from the propellers of the drone as suggested by a previous study [34]. The drone was operated in 30-min intervals at 8:00, 8:30, and 9:00 a.m. with a 40-s hovering at every altitude (i.e., 0, 10, 20, 30, 40, 50, 70, and 100 m) for the P1D and P2D periods. During the hovering time at each altitude, the first 20 s was regarded as an initial stabilization, and the measurement data for the following 20 s was averaged and used in the analysis.

The instruments used for stationary measurements were an optical particle counter (OPC) (Grimm Model 1.109A) for PM_10_, PM_2.5_, and PM_1_, mini aethalometer (Aethlab MA200) for BC, scanning mobility particle sizer (SMPS) (TSI SMPS3910) for nano-size particles, NO/NO_2_/NO_x_ analyzer (Eco Physics nCLD AL) for NO_x_, and CO_2_/O_3_ analyzer (KINSCO technology) for CO_2_ and O_3_ (Table 1). The inlet of every measuring instrument was installed outside approximately 1 m away from the apartment building wall to measure the outdoor air. Because the OPC only measures atmospheric particles with a diameter larger than 0.25 μm, the PM_2.5_ mass concentration was calculated by adding the mass concentration of smaller particles with a diameter between 11.5 and 205.4 nm measured by SMPS to the PM_2.5_ concentration measured by OPC. The PM_2.5_ concentration measured by AM520 used in the drone measurement tends to be higher than the PM_2.5_ concentration measured by OPC. Hence, the overestimated PM_2.5_ concentration measured by AM520 was fitted to the PM_2.5_ concentration measured by OPC at the same altitude.

### 2.3. WRF-CFD Modeling

**Figure 2:**
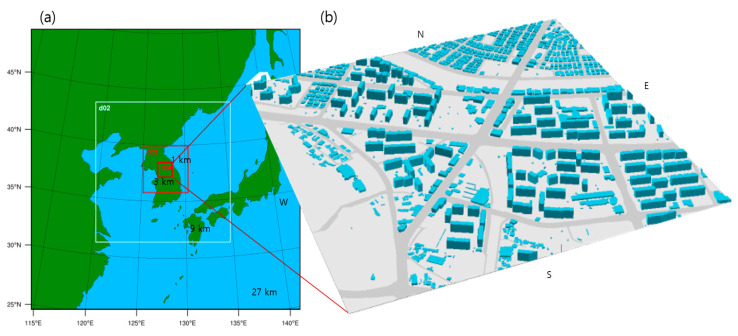
(**a**) Four nested domains in the WRF model and (**b**) a CFD model domain including the Toegye Intersection and the surrounding apartment and commercial buildings.

The coupled WRF-CFD model processes initial and boundary input data of the CFD model from the mesoscale meteorological fields (e.g., air temperature, wind direction, and wind speed) simulated by the WRF model [31]. The WRF model version 3.9.1 used in this study is a mesoscale meteorological model developed by the NCAR/UCAR (National Center for Atmospheric Research/University Corporation for Atmospheric Research, CO, USA). The simulation domain covers Northeast Asia and is centered at Chuncheon, Republic of Korea (37.85° N, 127.73° E). Four nested domains with horizontal grid sizes of 27, 9, 3, and 1 km were used (Figure 2a). The simulation periods were from 12 UTC on 10 to 12 March UTC on 15 March 2019 (P1) and from 00 UTC on 31 March 2019 to 00 UTC on 6 April 2019 (P2). The spin-up times were 35 and 23 h for P1 and P2, respectively. For the initial and boundary conditions, we used the National Centers for Environmental Prediction (NCEP) FNL reanalysis data with a 6 h interval and a horizontal resolution of 1° × 1°, the sea surface temperature data with a 24 h interval and a horizontal resolution of 0.5° × 0.5° archived by the National Oceanic and Atmospheric Administration (NOAA), and the United States Geological Survey (USGS) land cover data with a 1 km resolution. The planetary boundary layer (PBL) parametrization process greatly affects the accuracy of the near-surface air temperature and wind simulations. The Yonsei University (YSU) PBL scheme is a non-local PBL parameterization scheme that calculates the PBL height using the bulk Richardson number [35]. By contrast, the Mellor-Yamada-Janjic (MYJ) PBL scheme is a local PBL parameterization scheme that determines the vertical mixing through the calculation of turbulent kinetic energy (TKE), and thus calculates the PBL height from the TKE profile [36]. Therefore, the MYJ and YSU schemes were selected for simulation periods of P1 and P2, respectively, after examining the accuracy of the simulation results.

The CFD model is a Reynolds-averaged Navier–Stokes equations (RANS) model with a renormalization group (RNG) *k*–*ε* turbulent closure scheme, which simulates the atmospheric flow and pollutant dispersion in a staggered grid system based on the momentum equation, continuity equation, thermodynamic energy equation, turbulent kinetic energy and dissipation rate equations, and scalar transport equation [31,37]. The CFD model domain size is 1 km × 1 km × 473 m around the Toegye Intersection, with a horizontal grid size of 5 m × 5 m (Figure 2b). Vertical grid size is uniformly 3 m within the top floor of the apartment buildings and increases with an expansion ratio of 1.05 up to a vertical grid size of 13 m (i.e., maximum grid size). The total number of grids is 202 × 202 × 70. For surface boundary conditions, the digital topographic and building map of the National Geographic Information Institute was used, which includes the building floors and lane number information. The vertical profiles of air temperature, wind speed, and wind direction in the WRF model were linearly interpolated to provide the air temperature, wind speed, and wind direction data at every altitude up to 473 m in the CFD model domain. We conducted six CFD simulations at 8–9 a.m. on March 12, 14, and 15 and April 1, 2, and 3 in order to include the drone measurement periods. Each CFD simulation was run for 3600 s with a time interval of 0.5 s. The CFD simulation results were averaged from 2100 to 3600 s and used in the analysis. In simulating the NO_x_ concentration as a representative vehicle exhaust pollutant, every 5 × 5 × 3 m^3^ grid on the surface representing road with two or more lanes in the CFD model domain was set to emit NO_x_ based on the NO_x_ emission rate adopted from the CAPSS (Clean Air Policy Support System, National Institute of Environmental Research) (Table 2). In the NO_x_ emission calculation, the driving speed of 30 km h^−1^ was assumed and six types of vehicles (regular cars, SUVs, taxis, city buses, express buses, and heavy duty vehicles) were classified to calculate the NO_x_ emission factor. Then, the NO_x_ emission on road was estimated based on the traffic volumes of six vehicle types counted using the video records at the Toegye intersection. In the CFD simulations, NO_x_ is regarded as the no-reactive primary pollutant and has the zero background concentration to identify the 3-D spatial extent of near-road air pollutant focusing on traffic emission and dynamical and thermal dispersion [38,39].

## 3. Results and Discussion

### 3.1. Diurnal Variations of Near-Road Environments

In the diurnal variations of air temperature and relative humidity observed at the Chuncheon AWS, the lowest average air temperature was –2.9 ℃ (6 a.m.), and the highest average air temperature was 10.0 ℃ (2 p.m.), with a daily temperature difference larger than 10 ℃. The relative humidity exhibited an opposite trend to the air temperature. The diurnal variations of air temperature and relative humidity obviously followed their typical diurnal variation according to the surface heating and cooling. However, the relative humidity temporarily increased in the afternoon on March 12 and 15 because of light precipitation amount of 2–4 mm day^−1^. The predominant winds for the study periods were northerly and southwesterly winds. Geographically, Chuncheon is located in a basin with a low valley to the southwest and a high ridge to the north. Thus, the mountain-valley wind circulation on clear days in Chuncheon periodically produces a northerly wind at a low wind speed at night and a southwesterly wind at a high wind speed during the day [40,41]. The drone measurements were performed at 8–9 a.m. when the predominant winds were weak southerly-to-southwesterly winds with wind speeds less than 1.3 m s^−1^, except on March 15 (Table 3). Therefore, the drone and stationary measurement locations were located downwind of the Toegye Intersection, except on March 15. The urban background PM_2.5_, NO_2_, and O_3_ concentrations measured at the AQMS located approximately 1 km away from the intersection showed good or average air quality standard levels except on March 12. In summary, the background meteorological and environmental conditions for the study periods showed typical diurnal variations in spring season, allowing for the identification of near-road air pollution.

**Figure 3:**
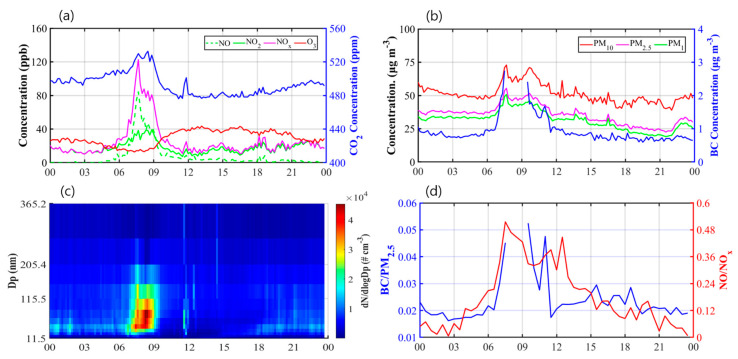
Time series of (**a**) gaseous pollutant (NO_x_, NO, NO_2_, O_3_, and CO_2_) concentrations, (**b**) particulate pollutant (PM_10_, PM_2.5_, PM_1_, and BC) concentrations, (**c**) size distributions of UFPs, and (**d**) concentration ratios (BC/PM_2.5_ and NO/NO_x_) measured at the stationary measurement location averaged over the P1D and P2D periods. The average time interval is 10 min except for the concentration ratios (30 min).

The diurnal variations of pollutant concentrations measured on the 11th floor of the apartment building for the P1D and P2D periods are shown in Figure 3. The averaged NO_x_ concentration was the highest at approximately 120 ppb during the morning rush hours (8–9 a.m.), which was 3.5 times higher than the daily-averaged NO_x_ concentration. During the morning rush hours, the concentration of NO primarily emitted from vehicles was over 80 ppb, accounting for most of the NO_x_. Because the NO emitted from the road reacts with O_3_ to form NO_2_, the O_3_ concentration during the morning rush hours was the lowest during a day [42]. The highest CO_2_ concentration was also shown during the morning rush hours coinciding with NO_x_ concentration, which is about 40 ppm higher than the lowest one.

The PM_10_, PM_2.5_, and PM_1_ also recorded the highest concentration during the morning rush hours. The highest PM_10_, PM_2.5_, and PM_1_ concentrations exceeded their daily averages by 23%, 35%, and 38%, respectively. Furthermore, the UFP number concentration measured by the SMPS during the morning rush hours was approximately three times higher than its daily average. The emitted particles from a diesel engine are initially UFPs with a diameter less than 100 nm. The emitted particles grow quickly in the atmosphere, and are mostly composed of PM_1_ within a few minutes [43,44]. The freshly emitted particles from a diesel engine are optically characterized as BC. Therefore, the BC similarly showed an increasing trend during the morning rush hours, and had a 3–6% proportion of PM_1_, even in the outdoor environment of the apartment building. As a result, the influence of the pollutants emitted on roads directly appeared at the stationary measurement location on 11st floor at 150 m away from the intersection.

In many measurement studies, analyses of concentration ratios between two pollutants have been utilized, both to minimize the effects of other environmental factors such as atmospheric stability and background concentration and to solely examine the effects of vehicle emissions [45,46]. The diurnal variations of NO/NO_x_ and BC/PM_2.5_ concentration ratios are presented in Figure 3d. The NO/NO_x_ and BC/PM_2.5_ ratios were 2.7 and at least 2.0 times higher, respectively, during the morning rush hours than their daily averages. Being coincident with the previous results, the effect of pollutants emitted on roads appeared especially large during the morning rush hours, regardless of other environmental factors. By contrast, the diurnal variations of all pollutant concentrations did not show similar increasing phenomena during the evening rush hours. This implies a relatively more effective atmospheric dispersion of emitted pollutants during the evening rush hours than during the morning rush hours, because the wind speeds in the afternoon were mostly higher than 2 m s^−1^ and the air temperature remained high until the evening hours.

### 3.2. Verification of WRF-CFD Model

The WRF simulation results were compared with the 1 h interval data observed at the Chuncheon AWS for morning hours (7–10 a.m.). The correlation coefficients between the WRF simulation results and observation data for the P1 period are 0.76 and 0.43 for air temperature and wind speed, respectively, whereas those for the P2 period are relatively higher (0.96 and 0.72 for air temperature and wind speed, respectively). The weather conditions for the P1 period included a couple of precipitation events and cloudy weather. By contrast, the weather conditions for the P2 period showed typically clear weather that continued for a week, which were suitable for an improved simulation performance of the WRF model. In previous studies verifying WRF simulations performed in China, Spain, and India, the correlation coefficients for air temperature and wind speed ranged between 0.78–0.94 and 0.20–0.53, respectively [47,48,49,50]. Their similar values to the results of this study demonstrate that the meteorological inputs provided from the WRF model to the CFD model are appropriate.

**Figure 4:**
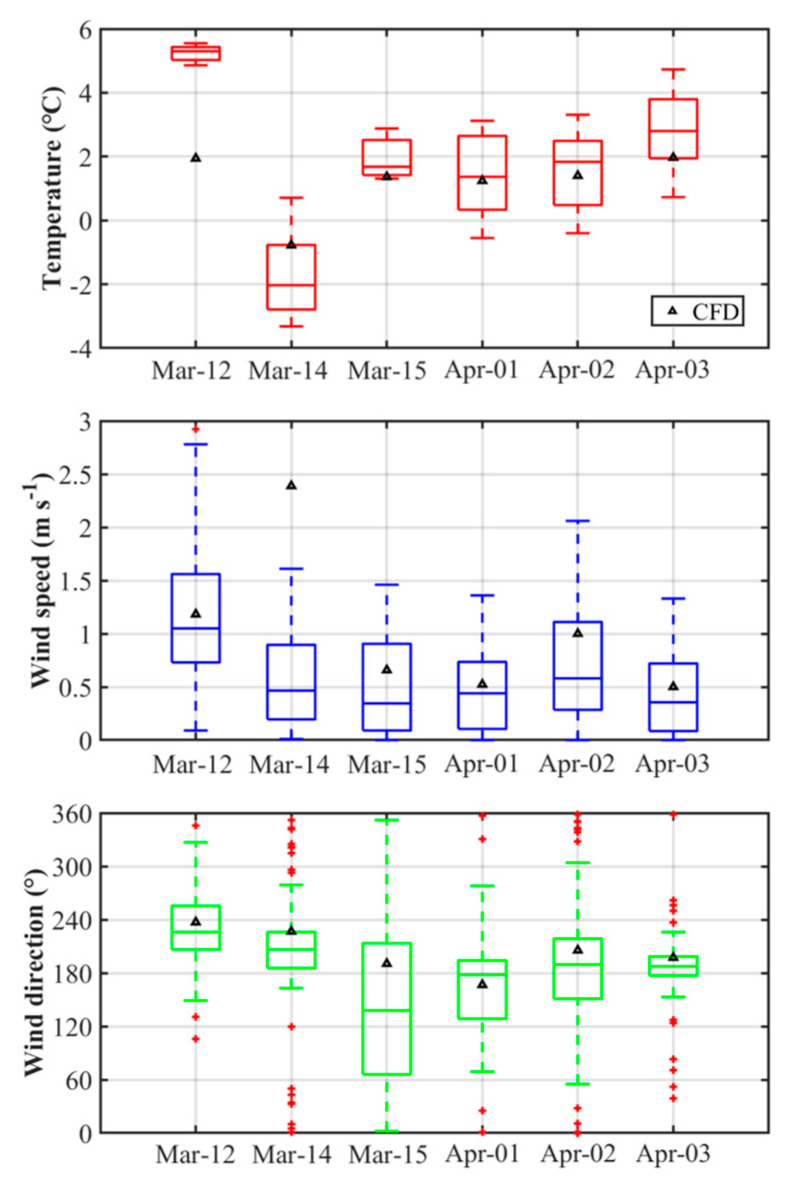
Boxplots of air temperature, wind speed, and wind direction of AWS observation data compared with CFD simulation results at the roadside location of Toegye Intersection. The number of data point is 60 min × 6 days (*n* = 360).

The air temperature, wind direction, and wind speed of the CFD simulation results during the morning rush hours were compared with the roadside AWS observation data with a 1-min interval at the Toegye Intersection for the P1D and P2D periods (Figure 4). The air temperature at the roadside location was underestimated in general, with a mean absolute error (MAE) of approximately 1 ℃. The simulated daily lowest air temperature gradually decreased and then increased after March 14, which was in accordance with the AWS observation data. The wind speed at the roadside AWS location was slightly overestimated with an MAE of 0.5 m s^−1^, showing the simulated wind speed similar to the observed wind speed, except for the strong wind case on March 14. The simulated wind direction at the roadside location was in accordance with the AWS observation data based on the sixteenth classification of wind direction. As a result, the CFD model properly simulated near-road meteorological fields that could reasonably represent air pollution distribution in the CFD model domain.

### 3.3. Spatial Distribution of Near-Road Environments

**Figure 5:**
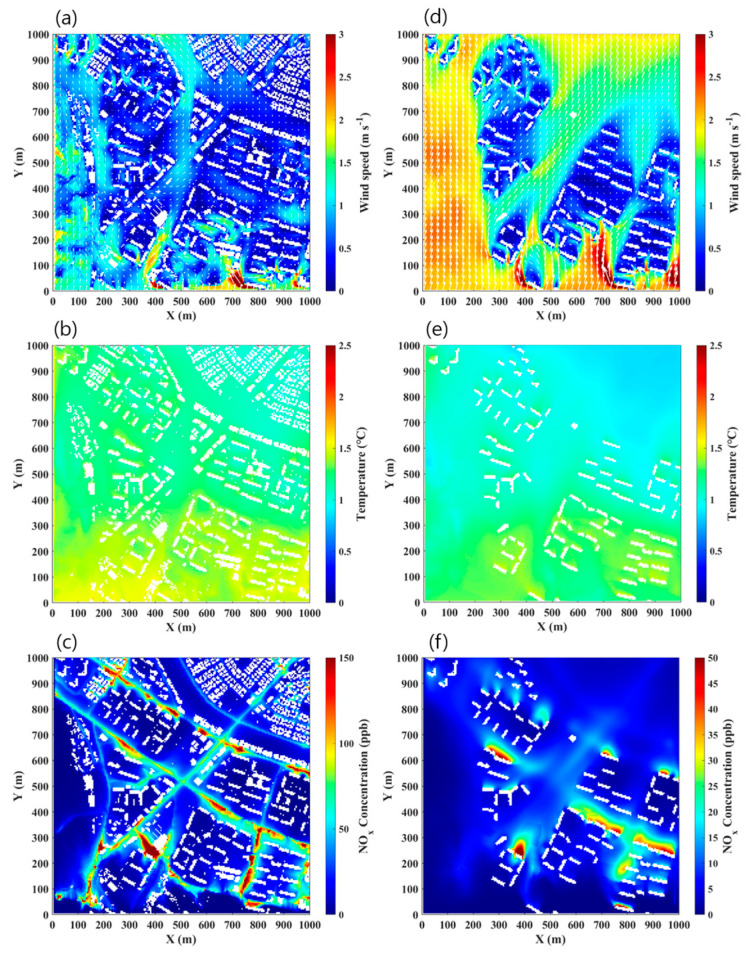
Simulated six-day averaged horizontal (**a**) wind speed, (**b**) air temperature, and (**c**) NO_x_ concentration fields at a 1.5 m altitude and horizontal (**d**) wind speed, (**e**) air temperature, and (**f**) NO_x_ concentration fields at a 30 m altitude during the P1D and P2D periods.

The simulated horizontal wind, air temperature, and NO_x_ concentration fields at the altitudes of 1.5 and 30 m are compared by averaging their distributions for six days during the P1D and P2D periods in total to identify the typical 3-D extent of traffic-related air pollution around the road intersection (Figure 5). Because of south-to-southwest winds during the study periods, the wind speed at the 1.5 m altitude was relatively high along the stream in the south and the urban park in the north of the intersection. By contrast, the wind speeds in the apartment complexes near the intersection were mostly lower than 1 m s^−1^ because of the blocking effect produced by the buildings (Figure 5a). The lower wind speeds in the apartment complexes also were shown at the 30 m altitude, which were clearly differentiated from the wind speeds in the rest of domain at the 30 m altitude (Figure 5d). The air temperatures tended to be higher in the apartment complexes compared to those in the rest of domain, while the air temperatures at the 30 m altitude were generally lower than those at the 1.5 m altitude within differences smaller than 0.5 ℃ depending on the location (Figure 5b,e). The NO_x_ concentrations at the 1.5 m altitude near the intersection and road were higher than those in the apartment complexes by 50 ppb or more (Figure 5c). The NO_x_ emitted from the road was more accumulated where the apartment and commercial buildings are elongated perpendicular to the wind direction near the road. The accumulated NO_x_ concentrations behind the buildings for the perpendicular wind direction to the road axis similarly appeared at the 30 m altitude (Figure 5f). The accumulation of NO_x_ emitted on roads is known as the street canyon effect with recirculating flows inside a street canyon especially with a high aspect ratio of building height to street width [51,52]. As a result, the NO_x_ distribution in three dimensions near the road intersection and apartment buildings was greatly influenced by mechanical factors such as geometries and arrangements of road and buildings rather than by thermal factors during the morning rush hours in presence of a weak solar heating on surfaces.

**Figure 6:**
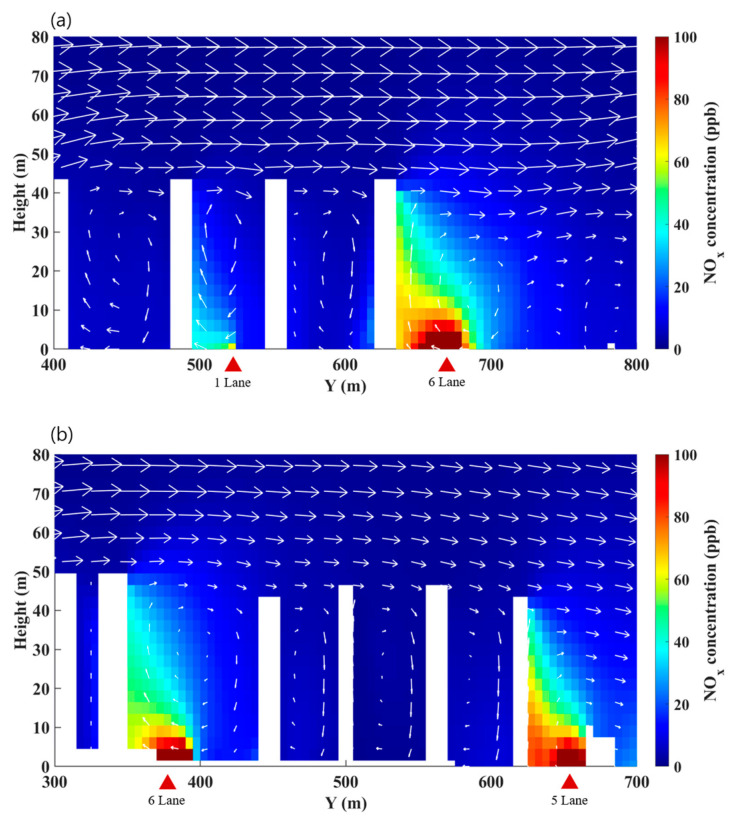
The *y*-*z* vertical cross-sections of simulated NO_x_ concentration fields at (**a**) *x* = 207 m and (**b**) *x* = 705 m. The arrows indicate the wind vectors of *v* and *w* components.

Figure 6 shows y–z vertical cross-sections at x = 207 m and x = 705 m where NO_x_ emitted on roads was apparently accumulated in street canyons. In the presence of the recirculating flows in street canyons as indicated by wind vectors, the emitted NO_x_ from mobile sources on roads is then transported upward to a higher floor along the leeward building walls. Similarly, previous studies [27,53] showed using CFD model simulations that the accumulation of emitted pollutant on roads gradually increases and then maximizes at a 3–10 m altitude from the ground near the roadside building walls because of the upward transport of emitted pollutant. In this study, compared to the previous studies, the maximized concentrations near the roadside building walls frequently appeared at a higher altitude because of taller building heights (i.e., approximately 50 m height). This leads to an increase in personal NO_x_ intake fraction for residents in the apartment buildings [54]. Consequently, high-rise roadside buildings worsen the near-road air quality by accumulating the emitted pollutants on roads not only at a ground level but also at elevated levels because of combination of the accumulation and upward transport along the roadside building walls. This clearly supports the analyzed results of stationary measurements at the 30 m altitude during the morning rush hours.

### 3.4. 3-D Spatial Extent of Near-Road Air Pollution

**Figure 7:**
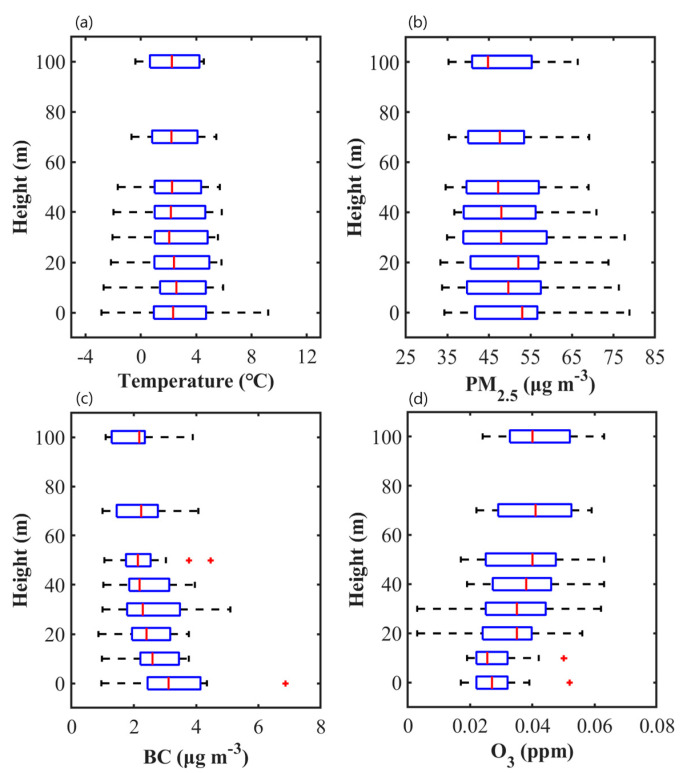
Vertical profiles of (**a**) air temperature, (**b**) PM_2.5_, (**c**) BC, and (**d**) O_3_ concentration boxplots for six days of P1D and P2D periods. A number of measurement data is 18 per each altitude.

We measured the vertical distribution of pollutant concentration using a drone and compared the measured result with the simulated one to support the 3-D spatial assessment of near-road air pollution. The measured profiles of air temperature, PM_2.5_, BC, and O_3_ concentrations during the morning rush hours for the P1D and P2D periods are shown in Figure 7. The air temperature profile exhibits that the near-surface atmosphere was relatively neutral because surface cooling during the nighttime transits to surface heating after sunrise. The stable-to-neutral atmospheric stability in the early morning is favorable for the accumulation of emitted pollutants near the surface. The PM_2.5_ concentration profile shows a decreasing trend as the altitude increases with the concentration median of 53 μg m^–3^ on the ground and 44 μg m^–3^ at the 100 m altitude. Most vertical variations in PM_2.5_ concentration appear below the apartment building height, while the PM_2.5_ concentration is almost uniform above the building height, which is similar to the previous study [55]. The decreasing trend is more apparent in the BC concentration profiles with the concentration median of 3.1 μg m^–3^ on the ground and 2.2 μg m^–3^ at the 100 m altitude. The steeper decrease in BC concentration than in PM_2.5_ concentration is attributed to the large proportion of BC in emitted particles especially in finer particles from vehicles on roads [56,57]. In contrast to PM_2.5_ and BC concentrations, the O_3_ concentration profile shows an increasing trend as the altitude increases with the concentration median of 0.027 ppm on the ground and 0.040 ppm at the 100 m altitude. This was a result of atmospheric O_3_ being actively titrated with NO_x_ emitted on roads. In summary, PM_2.5_, BC, and O_3_ all show significant vertical variations in their concentrations below the apartment building height at the downwind location of the road intersection in the urban park. This agrees well with the CFD simulation result showing the worsened air quality in the near-road environment surrounded by high-rise apartment buildings.

**Figure 8:**
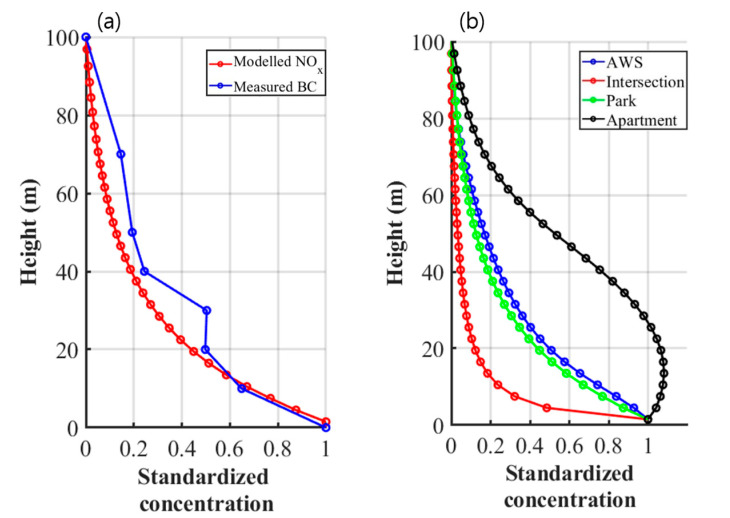
Standardized vertical profiles of (**a**) simulated NO_x_ and measured BC concentrations at the drone measurement location and (**b**) simulated NO_x_ concentrations at selected locations (i.e., AWS, Intersection, Park, and Apartment) around the road intersection.

BC and NO_x_ are commonly emitted from vehicles and have a strong correlation (R^2^ ≥ 0.78) in the measurement data during the morning rush hours [58]. Using the drone measurement data, therefore, we verify the accuracy of the CFD simulation results to identify the 3-D spatial extent of near-road air pollution around the intersection. To take the effect of on-road emission into account separately, the standardizing method is adopted for quantitatively comparing the measured BC and simulated NO_x_ concentration profiles to each other by setting them to 1 for the highest concentrations and to 0 for the lowest concentrations [59]. Figure 8a shows the standardized vertical profiles of measured BC and simulated NO_x_ concentrations at the drone measurement location for six days of the P1D and P2D periods. Both the measured BC and simulated NO_x_ concentrations sharply decrease with increasing altitude and are reduced by 50% approximately at the 20 m altitude compared to their highest concentrations on the ground. These similar decreasing trends of the measured BC and simulated NO_x_ concentrations guarantee the reliability of spatial distribution of NO_x_ concentration simulated by the CFD model.

The simulated NO_x_ concentration profiles at selected locations that are the road intersection (Intersection), roadside AWS location (AWS) with a 30 m distance from the intersection, the drone measurement location in the urban park (Park), and the stationary measurement location in the apartment building (Apartment) are shown in Figure 8b. The standardized NO_x_ concentrations at the ‘Park’ and ‘AWS’ locations show similarly decreasing profiles with increasing altitude, while that at the ‘Intersection’ location is almost reduced by 90% approximately at the 20 m altitude compared to the highest concentration on the ground. This is because the emitted NO_x_ at the intersection is mechanically transported horizontally rather than vertically in the presence of no or very weak surface heating on the ground. However, the standardized NO_x_ concentration at the ‘Apartment’ location shows its maximum approximately at the 15 m altitude rather than on the ground because the dispersion effect of plume transported downwind increases the plume height as the horizontal distance from the intersection increases. In addition, the upward transport of emitted NO_x_ along the apartment building wall also contributes to increase the plume height at the ‘Apartment’ location. As a result, the 3-D spatial extent of near-road air pollution is predominantly determined by the wind direction relative to the road axis and layout of roadside buildings.

**Figure 9:**
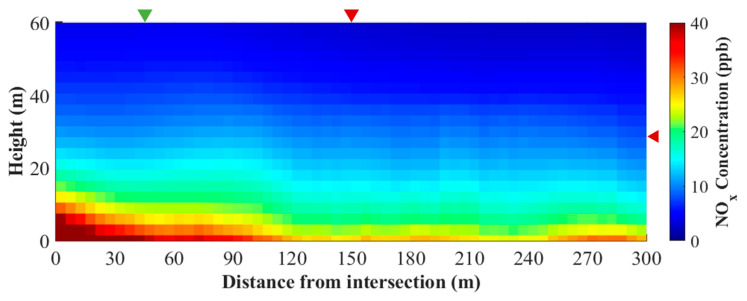
Composited cross-section of simulated NO_x_ concentration in terms of distances from the intersection at a model grid interval regardless of direction. Green and red markers denote the drone and stationary measurement locations, respectively.

To estimate the human exposure to traffic-related pollutants not only for pedestrians but also for residents in roadside apartment buildings, a representative 2-D vertical cross-section is composited in terms of the horizontal and vertical distances from the center of intersection using the simulated NO_x_ concentration regardless of direction (Figure 9). The composited NO_x_ concentration in the 2-D vertical cross-section is the highest at 76 ppb at the center of intersection, and then gradually decreases by 50% at a 50 m horizontal distance on the ground corresponding to the drone measurement location and by almost 90% at a 150 m horizontal and a 30 m vertical distance corresponding to the stationary measurement location of apartment building. The composited NO_x_ concentration at a certain altitude decreases, as the horizontal distance from the center of intersection increases up to a 120 m. Meanwhile, the composited NO_x_ concentration shows secondary peaks at the horizontal distances of approximately 200 m and 270 m from the center of intersection which is sufficiently far from the intersection because of NO_x_ emission on nearby roads. In conclusion, the horizontal extent of near-road air pollution around the intersection is at least 120 m or, to some directions, 200 m from the center of intersection. The vertical extent of near-road air pollution around the intersection is at least a half of roadside building height or possibly higher up to the roadside building height in the downwind direction.

## 4. Conclusions

The 3-D spatial extent of the near-road air pollution around the intersection in an urban residential area was investigated using two complementary methods that are drone monitoring and WRF-CFD modeling. Based on the stationary measurement data collected on 11th floor of the roadside apartment building (i.e., 30 m height), we focused on the morning rush hours when the measured concentrations of traffic-related air pollutants such as NO_x_, BC, and UFP were maximized during a day. The WRF-CFD model simulated the NO_x_ concentration fields around the intersection during the morning rush hours. The simulated highest concentration appeared on roads between roadside apartment buildings, which was attributed to the accumulation of emitted NO_x_ following recirculating flows inside street canyons when the prevailing wind direction was almost perpendicular to the street canyon. The drone measurement conducted close to the road intersection showed decreasing trends in the measured BC and PM_2.5_ concentrations with increasing altitude. The vertical gradient of measured BC concentration was steeper than that of measured PM_2.5_ concentration because BC has a large portion in emitted particles from vehicles. The vertical concentration gradients of all measured pollutants including BC, PM_2.5_, and O_3_ became insignificant at altitudes higher than the apartment building height (i.e., 50 m height), implying the weakened pollutant dispersion and worsened air quality in building canopies. The standardized BC concentration measured using the drone and NO_x_ concentration simulated using the WRF-CFD model were inter-compared and matched closely with each other especially near ground levels. Consequently, the spatial extents of near-road air pollution adversely affecting the health of pedestrians and residents covered at least 120 m distance from the center of intersection horizontally and at least a half of building height vertically, but in some cases, up to the nearby road network and to the top floor of building. More importantly, the 3-D spatial extent of near-road air pollution was determined by the atmospheric transport governed by the building arrangement and prevailing winds in the urban residential area.

The estimated spatial extent of air pollution using drone monitoring and WRF-CFD modeling is a site-specific result that can be further extended in other urban residential areas with larger traffic emissions and more complicated building geometries. In this study, it is assumed that there is no pollutant emission in the CFD model domain other than road traffic, which is idealistic condition. In a complicated atmospheric environment, various pollutant sources, such as residential heating, industrial complexes, and biomass burning, need to be incorporated for assessing the realistic spatial distribution of air pollutants. The main achievement of this study is, first, the comparability of two complementary methods for examining the spatiotemporal variabilities of local-scale air quality. In addition, the standardized measured and simulated concentrations can be further interpreted as representative local air quality profiles near air pollution hotspots, such as main roads and intersections, for planning and assessing environmental policy on urban air quality.

## Figures and Tables

**Table 1:** Specifications of instruments used for drone and stationary measurements.

	Variable	Model and Manufacturer	Measurement Resolution	Accuracy	Time Interval
Drone	PM_2.5_	Sidepak AM520, TSI (MN, USA)	1 μg m^−3^	-	1 s
	Black carbon (BC)	MA200, Aethlab (CA, USA)	0.001 μg m^−3^	-	5 s
	O_3_	Aeroqual 500, Aeroqual (Auckland, NZ)	0.001 ppm	±0.008 ppm	1 min
	Air temperature Relative humidity (RH)	Imet-XQ_2_, InterMet (MI, USA)	0.01 ℃, 0.1%	±0.3 ℃, ±5%	1 s
Stationary	PM_10_, PM_2.5_, PM_1_	Model 1.109A, Grimm (NC, USA)	0.1 μg m^−3^	±5%	6 s
	Particle number	Nanoscan SMPS3910, TSI (MN, USA)	100 particles cm^−3^	-	1 min
	BC	MA200, Aethlab	0.001 μg m^−3^	-	1 min
	NO_x_, NO_2_, NO	nCLD AL, Eco Physics (Duernten, Switzerland)	0.001 ppm	-	1 s
	CO_2_	CO_2_ analyzer, KINSCO technology (Seoul, ROK)	1 ppm	-	1 min
	O_3_	O_3_ analyzer, KINSCO technology (Seoul, ROK)	0.001 ppm	-	1 min

**Table 2:** NO_x_ emission factors classified by vehicle type in CAPSS (*v* = driving speed).

Vehicle Type	Fuel	Emission Factor (g km^−1^)
Regular car	Gasoline	$\left(-3.5\times 10^{-6}\right)v^{2}+0.00033v+0.0112$
SUV, Light truck	Diesel	$14.2\times v^{-0.7747}$
Taxi	LPG	$-0.000004v^{2}+0.0006v+0.0055$
City bus	CNG	$8.6972\times e^{-0.0130v}$
Express bus	Diesel	$40.9398\times v^{-0.5611}$
Heavy duty vehicle	Diesel	$107.5\times v^{-0.5679}$

**Table 3:** Daily meteorological and urban air quality data during the morning rush hours (8–9 a.m.) for the P1D and P2D periods. The wind direction increases clockwise from 0° (i.e., due north). The daily precipitation amounts are indicated together with the precipitation time.

		Temp. (℃)	Wind Speed (m s^−1^)	Wind Direction (°)	Daily Precipitation (mm)	Cloud Cover (1/10)	PM_2.5_ (μg m^−3^)	NO_2_ (ppb)	O_3_ (ppb)
P1D	Mar-12	5.3	1.3	228	2.4 (10 a.m.–4 p.m.)	9	45	21	37
	Mar-14	−1.7	0.6	217	-	0	23	32	7
	Mar-15	1.9	0.5	97	4.0 (3–11 p.m.)	10	30	14	22
P2D	Apr-01	1.5	0.5	164	-	0	23	30	10
	Apr-02	1.6	0.7	207	-	0	22	8	28
	Apr-03	2.8	0.4	189	-	0	23	12	28
